# Oral Conditions as Risk Factors for Low Oral Health-Related Quality of Life Among the Elderly Population in Yogyakarta, Indonesia

**DOI:** 10.1055/s-0042-1757566

**Published:** 2022-12-13

**Authors:** Dewi Agustina, Lisdrianto Hanindriyo, Bernadetta Esti Chrismawaty, Fimma Naritasari

**Affiliations:** 1 Department of Oral Medicine, Faculty of Dentistry, Universitas Gadjah Mada, Yogyakarta, Indonesia; 2 Department of Preventive and Community Dentistry, Faculty of Dentistry, Universitas Gadjah Mada, Yogyakarta, Indonesia

**Keywords:** dental occlusion, quality of life, xerostomia

## Abstract

**Objective** Approximately 70% of the elderly population living in Yogyakarta, Indonesia, has a low oral health-related quality of life (OHRQoL). This study aimed to identify the risk factors for low OHRQoL in the elderly population of Yogyakarta.

**Materials and Methods** Oral Hygiene Index Simplified (OHI-S), Community Periodontal Index, Decayed, Missing, and Filled Teeth (DMFT) Index, and the number of natural occluding pairs (NOP) were assessed for 153 participants aged ≥ 60 years. Xerostomia, hyposalivation, and OHRQoL were also examined using Xerostomia Inventory (XI), unstimulated spitting whole saliva collecting method, and Geriatric Oral Health Assessment Index (GOHAI) questionnaire, respectively. OHRQoL was categorized as low, moderate, and high.

**Statistical Analysis** Bivariate and multivariate tests were conducted to identify the risk factors for low OHRQoL.

**Results** Initial analysis of characteristics of participants revealed that hyposalivation, xerostomia, periodontal pocket, high DMFT, NOP ≤ 5, poor OHI-S, and low OHRQoL were experienced by 40 (26.1%), 92 (60.1%), 39 (25.5%), 110 (71.9%), 112 (73.2%), 44 (28.8%), and 108 (70.6%) participants, respectively. Relative risk (RR) and *p* values for hyposalivation, xerostomia, periodontal pocket, high DMFT, NOP ≤5, and poor OHI-S were found to be at 1.573 (CI 0.681–3.637) and 0.225; 2.532 (CI 1.255–5.108) and 0.006; 0.846 (CI 0.391–1.830) and 0.606; 1.759 (CI 0.843–3.670) and 0.110; 1.133 (CI 0.522–2.461) and 0.008; and 2.723 (CI 1.293–5.734) and 0.632, respectively. Multivariate tests showed that xerostomia and NOP ≤5 had RR of 2.519 (CI 1.221–5.195) and 2.536 (CI 1.175–5.477), respectively.

**Conclusions** Overall, elders with xerostomia or NOP ≤ 5 had 2.5 times higher risk of having a low OHRQoL.

## Introduction

Thus far, quality of life assessment is mostly based on the general health of people. 1 2 Oral health might play a considerable role in determining life quality. Quality of life itself cannot be separated from the overall condition of a human being, which comprises general and oral health. 3 Health and function of the mouth deteriorate with age. 4 Poor oral conditions among elderly people are particularly influenced by edentulism, dental caries, periodontal disease, xerostomia, salivary gland dysfunction, and oral mucosal lesions, including premalignant lesions. 5 6 These conditions severely impact the daily life of elderly people and decrease their oral function, self-confidence, and social life. This decline eventually affects the oral health-related quality of life (OHRQoL), 5 7 which reflects the satisfaction of people with respect to oral functions, such as eating, sleeping, and engaging in social interaction, as well as their self-esteem. 8 OHRQoL is assessed by studying how factors such as function, pain, and psychological and social aspects affect the well-being of an individual. 9

This type of research is essential in Indonesia because Indonesian populations can be categorized into old age groups; the percentage of elders reached 7% in 2010 and will continuously increase up to 14.1% in 2030 10 11 and 15.8% (an increase of 8.2%) by 2035. 12 The number of Indonesian elders is predicted to become the highest in the world. 13 Yogyakarta is one of the provinces in Indonesia with the highest number of elders. Around 14% of the populations in Yogyakarta are elders. 10 12 Yogyakarta is also a city with the longest life expectancy in Indonesia; therefore, many elderly associations are rapidly growing in this area. 11 12 A previous preliminary study demonstrated that around 70% of elders in Yogyakarta had low OHRQoL.

Old age may contribute to several health problems either as a result of physiologic (degenerative) or pathologic processes. Elderly people may be susceptible to acute or chronic disease due to their decreasing immune system, thus making them consume additional medications. Deterioration of physiologic condition, polypharmacy, and the high occurrence of chronic diseases in elders may manifest in unavoidable oral cavities. 14 The only possible approach is to minimize their effects to help elders increase their quality of life regardless of their health condition.

Xerostomia (dry mouth) either with or without hyposalivation is one of the common oral manifestations complained about by elders. Reports indicate that 80% of prescribed medications cause xerostomia. 15 16 17 18 Xerostomia and hyposalivation easily contribute to the development of poor oral hygiene, dental caries, and periodontal disease due to the reduced function of saliva. 17 18 Dental caries and periodontal disease are the main causes of tooth loss. Thus, tooth loss without rehabilitative treatment results in mastication impairment, which, in turn, affects OHRQoL. A previous study in Yogyakarta found that 69.85% of the elderly had poor oral OHRQoL (2013, unpublished research). From the study was found as well that the majority of elderly oral health was poor, but when the OHRQoL assessment was carried out, it turned out that the OHRQoL was still satisfactory. For this reason, it is very important identifying oral conditions that may be potential risk factors for the low OHRQoL of elders in Yogyakarta, which was the aim of this study. The implications of this study will help the government in planning and improving oral health management for elders, which is expected to increase their OHRQoL.

## Materials and Methods

### Participants

This observational cross-sectional study involved 153 participants (≥60 years) comprising 39 males and 114 females from five elderly associations ( *Posyandu Lansia* ) from three subdistricts in Yogyakarta with a high number of elderly (Danurejan, Gedongtengen, and Jetis). The five *Posyandu Lansia* (Kemetiran, Gowongan, Suryatmajan, Tegalpanggung, and Danurejan) were randomly chosen from these subdistricts. Inclusion criteria of participants: people who are ≥60 years of age and can hear and communicate well.

To recruit the participants, forty letters of invitation to this survey were mailed for each *Posyandu Lansia* , so 200 letters of invitation were addressed to people aged 60 years and over. All recipients were informed about the aim and study course. Appointment for the examination was given for individuals upon submission of their written informed consent as their agreement to be involved in this research. There were 176 positive responders, after the invitation. Eighteen persons of 176 positive responders did not come to the examination venue due to some reasons, i.e., 8 persons with urgent personal matters and 10 persons with obstacles in transportation. The other five persons came to the examination venue but with incomplete data due to incomplete questionnaire filling because of difficulty in communicating, listening, and capturing questions. Finally, there were 153 participants involved in this research as study subjects. None of the participants required special assistance for their daily activities.

Ethics approval for conducting this study has been obtained from the Ethics Committee of the Dentistry Faculty, Universitas Gadjah Mada, Yogyakarta, Indonesia (Approval number: 683/KKEP/FKG-UGM/EC/2014).

### Measurements

The participants underwent intraoral examination to assess oral hygiene (OH), periodontal tissue condition, DMFT index (the number of decayed, missing, and filled teeth), and the number of natural occluding pairs (NOP) of teeth.

Oral hygiene was determined using OHI-S developed by Greene and Vermillion with the aid of the no. 5 explorer that was moved along the examined tooth surface. The OH score was classified as 0.0–1.2 (good), 1.3–3.0 (fair), and 3.1–6.0 (poor). The condition of the periodontal tissue was evaluated using the WHO periodontal probe with a round tip of 0.5 mm diameter. The Community Periodontal Index of the WHO was applied. The score of the periodontal tissue condition was classified into the following: 0 (healthy tissue), 1 (bleeding of probing), 2 (calculus with a plaque by probing), 3 (4–5 mm of pathologic pocket), and 4 (≥6 mm of pathologic pocket). The status of periodontal tissue each tooth was determined according to the highest classification. The number of DMFT was determined using the DMFT index, and the examination was clinically performed using dental diagnostic instruments. The number of NOP of teeth was also clinically examined and counted. Each examination helped determine the oral health status of the participants and was used as an oral health status indicator.

OHRQoL was determined using GOHAI. 19 The 12-item questionnaire of GOHAI was developed in the Indonesian version after validity and reliability tests were done ( *r* = 0.287, *p* < 0.05; Cronbach's alpha coefficient = 0.783) to assess the following three dimensions of OHRQoL: physical function, pain or discomfort, and psychosocial function. This questionnaire comprises a six-point Likert scale from never, rarely, occasionally, fairly often, very often to always. The final score ranges from 0 to 60, wherein a high score indicates an improved OHRQoL. The GOHAI score was classified as high (57–60), moderate (51–56), and low (≤50). Xerostomia was determined by interviewing participants about the history of dry mouth in the past three months on the Xerostomia Inventory (XI) in the Indonesian version after validity and reliability tests were done ( *r* = 0.295, *p* < 0.05; Cronbach's alpha coefficient = 0.890), which comprises an 11-item questionnaire covering experiential and behavioral aspects of xerostomia. The questionnaire comprises a five-point Likert scale from never, hardly ever, occasionally, fairly often to very often. 20 Xerostomia was determined when participants answered “fairly often” and/or “very often”. 21 Hyposalivation (volume of saliva <0.1 ml/min) was identified by measuring whole unstimulated saliva flow using the spitting method. In this study, measurement of DMFT and oral hygiene were conducted by FN, and measurement of periodontal status and NOP were conducted by BEC for all participants. Four professional students of the Faculty of Dentistry Universitas Gadjah Mada helped FN and BEC in writing the measurement results in Oral Health Status Forms. Filling in the questionnaires of GOHAI and XI by interviewing all participants was carried out by DA. Measuring salivary flow for all participants was done by DA with the assistance of other four professional students of the Faculty of Dentistry, Universitas Gadjah Mada to weigh the saliva and write the measurement results in Oral Health Status Forms. Measuring the salivary flow was conducted from 8 a.m. to 10 a.m.

### Statistical Analysis

For statistical analyses, the results of variable measurements were categorized as low OHRQoL if the GOHAI score of ≤50, and high OHRQoL if the GOHAI score > 50. Xerostomic status was categorized as xerostomia and non xerostomia. Hyposalivation status was categorized as hyposalivation and nonhyposalivation. Oral hygiene status was categorized as poor oral hygiene if the OHI-S score of ≥3.1, and good oral hygiene if the OHI-S score < 3.1. NOP status was categorized as NOP ≤5 and NOP >5. DMFT Index was categorized as high if the DMFT Index >13.9 and low if the DMFT Index ≤13.9. The periodontal pocket was determined if the classification of CPI were 3 and 4, meanwhile periodontal status was determined not having a periodontal pocket if the classification of CPI were 0, 1, and 2.

Bivariate and multivariate analyses were conducted using SPSS software 16.0 version (IBM Corp., Chicago) to estimate the risk factors for low OHRQOL.

## Results

The results from the subject's characteristics based on OHRQoL in Table 1 demonstrated that only gender (p = 0.024) and occupation (p = 0.028) had significant contributions to the proportion of the OHRQoL status. Female subjects had a more significant proportion of poor OHRQoL status compared to male subjects. Formal worker subjects had a more significant proportion of poor OHRQoL status compared to the other two occupations (household worker and informal worker).

**Table 1:** Subject's characteristics based on OHRQoL ( *N* = 153)

No.	Variable	OHRQoL		*p* -Value*
		*n* (%)				
		Poor	Good	
1.	Gender			**0.024**
	Male	22 (20.4)	17 (37.8)	
	Female	86 (79,6)	28 (62.2)	
2.	Education			0.786
	Non-formal education	31 (28.7)	10 (22.2)	
	Elementary school	20 (18.5)	10 (22.2)	
	Junior high school	37 (34.3)	14 (31.3)	
	Senior high school	13 (12)	6 (13.3)	
	University/college	7 (6.5)	5 (11.1)	
3.	Residential			0.476
	Rural	30 (27.8)	10 (22.2)	
	Urban	78 (72.2)	35 (77.8)	
4.	Ethnicity			0.153
	Javanese	107 (99.1)	43 (95.6)	
	Non-Javanese	1 (0.9)	2 (4.4)	
5.	Marital Status			0.779
	Widowed	61 (56.5)	28 (62.2)	
	Married	45 (41.7)	16 (35.6)	
	Not-married	2 (1.9)	1 (2.2)	
6.	Occupation			**0.028**
	Household worker	39 (36.1)	11 (24.4)	
	Informal worker	13 (12)	1 (2.2)	
	Formal worker	56 (51.9)	33 (73.3)	
7.	Systemic disease			0.983
	No systemic disease	53 (49.1)	22 (48.9)	
	Systemic disease	55 (50.9)	23 (51.1)	
8.	Medication			0.500
	No routine medication	71 (65.7)	27 (60)	
	Routine medication	37 (34.3)	18 (40)	

Abbreviations: GOHAI, Geriatric Oral Health Assessment Index; OHRQoL, oral health-related quality of life.

*Chi-square analysis.

As depicted in Table 2 , the means of OHIS, GOHAI, DMFT, NOP, and salivary flow rate measurement were categorized as moderate, poor, high, ≤5, and normal, respectively. The percentage of subjects with periodontal pockets was less than in subjects without a periodontal pocket. In contrast, the percentage of subjects with xerostomia was more than in subjects without xerostomia.

**Table 2:** Means and percentages of variable measurement ( *N* = 153)

No.	Risk factors	Mean ± SD
1.	OH	2.31 ± 1.36
2.	GOHAI	45.5 ± 9.52
3.	DMFT	20.13 ± 9.45
4.	NOP	2.93 ± 4.03
5.	Salivary flow rate (mL/min)	0.26 ± 0.26
		***n*** **(%)**
6.	Periodontal pocket	
	Yes	39 (25.5)
	No	114 (74.5)
7.	Xerostomia	
	Yes	92 (60.13)
	No	61 (39.87)

Abbreviations: DMFT, decayed, missing, and filled teeth; GOHAI, Geriatric Oral Health Assessment Index; NOP, natural occluding pairs; OH, oral hygiene; SD, standard deviation.

The results showed that participants with NOP ≤5 have the highest percentage of 79.4%, followed by high DMFT (75.7%) and xerostomia (67.3%) as shown in Table 3 . Participants with low OHRQoL were found to be as much as 70.6%.

**Table 3:** Frequency for each risk factor related with low OHRQoL ( *N* = 153)

		Low OHRQoL	
No.	Risk Factors	Yes *n* (%)	No *n* (%)
1.	Hyposalivation		
	Yes	31 (29)	9 (19.6)
	No	76 (71)	37 (80.4)
2.	Xerostomia		
	Yes	72 (67.3)	20 (43.5)
	No	35 (32.7)	26 (56.5)
3.	Periodontal pocket		
	Yes	26 (24.3)	13 (28.3)
	No	81 (75.7)	33 (71.7)
4.	High DMFT		
	Yes	81 (75.7)	29 (63)
	No	26 (24.3)	17 (37)
5.	NOP ≤ 5		
	Yes	85 (79.4)	27 (58.7)
	No	22 (20.6)	19 (41.3)
6.	Poor OH		
	Yes	32 (29.9)	12 (26.1)
	No	75 (70.1)	34 (73.9)

Abbreviations: DMFT, decayed, missing, and filled teeth; NOP, natural occluding pairs; OH, oral hygiene; OHRQoL, oral health-related quality of life.

Bivariate analysis was conducted to determine the size of the association strength of each risk factor with low OHRQoL. This analysis revealed that xerostomia and NOP ≤5 had a significant relationship with low OHRQoL as shown in Table 4 . Subsequently, two risk factors (xerostomia and NOP ≤5), which had a p < 0.05, were involved in the multivariate analysis.

**Table 4:** Bivariate analysis to measure the association strength among each risk factor with low OHRQoL

No.	Risk Factors	*p* -Value	*r*	CI (95%)
1.	Hyposalivation	0.225	1.573	0.681–3.637
2.	Xerostomia	0.006*	2.532	1.255–5.108
3.	Periodontal pocket	0.606	0.846	0.391–1.830
4.	High DMFT	0.110	1.759	0.843–3.670
5.	NOP ≤5	0.008*	1.133	0.522–2.461
6.	Poor OH	0.632	2.723	1.293–5.734

Abbreviations: CI, confidence interval; DMFT, decayed, missing, and filled teeth; NOP, natural occluding pairs; OH, oral hygiene; OHRQoL, oral health-related quality of life; *r* , correlation coefficient.

*) *p* < 0.0.

The results from the multivariate analysis in Table 5 indicated that xerostomia and NOP ≤5 were the risk factors for low OHRQoL in the elders living in Yogyakarta. Participants with xerostomia or NOP ≤5 had 2.5 times higher risk of having low OHRQoL.

**Table 5:** Multivariate analysis of xerostomia and NOP ≤5 toward low OHRQoL

			95% CI for OR	
Risk factors	Sig.	OR	Lower	Upper
Xerostomia	0.012	2.519	1.221	5.195
NOP ≤5	0.018	2.536	1.175	5.477

Abbreviations: CI, confidence interval; NOP, natural occluding pairs; OHRQoL, oral health-related quality of life; OR, odds ratio; Sig., significance.

## Discussion

Table 1 demonstrated that only gender and occupation had significant contributions to the proportion of the OHRQoL status. Female subjects had a more significant proportion of poor OHRQoL status compared to male subjects. Formal worker subjects had a more significant proportion of poor OHRQoL status compared to the other two occupations (household worker and informal worker). Gender was also considered a factor influencing OHRQoL. Cohen-Carneiro et al. clearly show that women are associated with a negative perception of OHRQoL. 22 Women will be more worried, disturbed, and dissatisfied with their oral health, even though women actually have more knowledge about oral health than men. 23 For this reason, when women are asked to rate their quality of life, they tend to rate lower because in general their standards and expectations are higher. Their review showed that there is a positive relationship between the level of poverty and education with OHRQoL. 22 The poorer or the lower the education of a person, the worse the impact on OHRQoL. What was conveyed by Cohen-Carneiro et al. is not in line with the results of this study. Poor OHRQoL is more common in formal workers than in households and informal workers. Formal workers usually come from people who are more educated. As it is known, a person who is more educated tends to judge something with a more complex and careful perception because it is based on his broader knowledge and many considerations. When they are asked to rate their quality of life, they tend to rate very carefully or lower.

Xerostomia or dry mouth is a subjective sensation experienced by approximately 20% of adults. 24 25 The prevalence of xerostomia increases with age. Approximately 30% of the population aged ≥65 years have experienced xerostomia. 20 24 The xerostomia percentage of participants with low OHRQoL was 67.3% in this study. Xerostomia can also be accompanied with or without hyposalivation. Similarly, hyposalivation is not always accompanied by xerostomia. The causes of xerostomia are quite varied, ranging from the effects of uncontrolled systemic diseases, such as diabetes mellitus, to the effect of medications, such as antihypertensive drugs, radiotherapy and chemotherapy complications, dehydration, and certain psychological conditions (such as anxiety and stress). 24 25 26

The results of this study demonstrated that 90.1% of subjects experienced xerostomia, however, only 26.1% were proven to be hyposalivation. This condition was probably contributed by 18 subjects with diabetes mellitus and 42 subjects with hypertension, most of whom were taking antihypertensive drugs which were xerogenic. Other xerogenic drugs that might affect xerostomia include antidepressants, antihistamines, cytotoxics, and bronchodilators. 18 26 Approximately 500 xerogenic drugs from 42 drug categories and 56 subcategories were also found. 27 A total of 80% of the drugs prescribed by doctors have an effect on xerostomia in the elders. 15 28 Therefore, assuming that the medications taken by the elders might play an important role in the development of xerostomia or hyposalivation is reasonable. 16 18 Moreover, assuming that the elders involved in this study were taking other xerogenic drugs that might affect the prevalence of xerostomia is justifiable. Participants with xerostomia in this study might experience hyposalivation given that the prevalence of hyposalivation in this study reached 26.1%. This prevalence is lower compared with the meta-analysis report of hyposalivation prevalence for unstimulated methods in elderly people (33.39%). 29 Saliva has many important roles, including its buffering capacity function, antibacterial activity, the self-cleansing mechanism of the liquid component, and its function related to food intake. 30 31 32 33 Therefore, reduced salivary flow decreases the ability of the saliva to perform its vital functions, such as self-cleansing mechanism, antibacterial action, neutralization of its pH in the oral cavity, and tooth remineralization. 32 33 By contrast, two diseases demonstrated the highest prevalence in the oral cavity, namely, dental caries and periodontal disease, which is an infectious disease. Reduction in salivary flow might lead to impaired abilities to improve oral hygiene and prevent periodontal disease and dental caries. 34 35 This finding is proven by the high percentage of DMFT, periodontal pockets, and poor OH among the participants in this study. Tooth loss can be the endpoint of these diseases if left untreated. Teeth have several vital functions, such as masticatory function, speech production, and aesthetics. These functions could not be optimally performed in the absence of several teeth.

The various effects of xerostomia, such as poor oral hygiene, dental caries, and NOP ≤5, disturb the function and comfort of the oral cavity. The percentages of the xerostomia effects in this study were relatively high. This finding is quite rational because it impacted the low OHRQoL. Moreover, a previous study has shown that poor oral hygiene leads to a low OHRQoL. 36 Another study also demonstrated the presence of a negative moderate correlation between the occurrence of dental caries and OHRQoL among the elderly population in Yogyakarta. 37 In their systematic review, Rijt et al. stated that OHRQol was positively correlated with a higher number of teeth and NOP, but negatively correlated with xerostomia, orofacial pain, and poor chewing ability. 38 Rijt et al.'s statement corroborates the results of this study which indicates that xerostomia and a small amount of NOP are risk factors for low OHRQoL.

The assessment of xerostomia and OHRQoL in this study is subjective because it depends on the perception of a person. Locker argued that the concept of quality of life is elusive and abstract. 39 Intuitively, life quality is understandable but is difficult to define. 1 2 Perceptions of life quality can also be influenced by many factors, such as social conditions, level of education, culture, politics, and practice, depending on the place where the concept is applied and measured. The analysis is closely related to values of life, which substantially vary from one person to another. 2 5 40 Petersen and Yamamoto indicated that life quality is one's perception of life considering culture and norms based on a person's life related to the goals, expectations, standards, and concerns over their lifetime. 41 Thus, OHRQoL is simply defined as how good is your mouth for you. OHRQoL assessment is subjective; thus, as a reasonable risk factor for low OHRQoL rather than hyposalivation, xerostomia assessment is also subjective. This finding was quite plausible based on a previous study, which revealed hyposalivation as the risk factor for poor oral health status; thus, both assessments are objective. 42 The results of this study are in accordance with the results of research by Arsyad and Syamson stated that the elderly who experienced xerostomia had a lower OHRQoL as measured by OHIP-14 than the elderly who did not experience xerostomia. 43 This indicates that the results of this study are still relevant to the condition of the elderly lately. The relevance of the results of this study to recent conditions is also supported by the percentage of poor OHRQoL in elderly subjects from the study of Gita et al. (66.7%). 44 Gita et al.'s result is not much different from the percentage of poor OHRQoL in this study (70.1%). The OHRQoL measurement instrument used is the same, namely GOHAI.

Considering the effects of xerostomia and NOP ≤5, the participants will reasonably have low OHRQoL due to disturbances in some aspects of function, pain/discomfort, psychological conditions, and disrupted social well-being. One way to increase OHRQoL is by wearing dentures. This suggestion is based on previous research which proved that the use of dentures in elderly patients who lost their teeth partially or completely can increase OHRQoL, reduce anxiety, and feel more saliva secretion. 45 The lack of salivary secretion might also cause a variety of disorders in the oral cavity, such as poor oral hygiene, dental caries, periodontal disease, and burning sensation, which will eventually induce pain. Disruption of mastication perceived by the individuals from tooth loss might increase the possibility of an individual withdrawing from social interactions. This phenomenon will affect the psychological condition of a person that can impact OHRQoL.

Saliva forms food boluses to ease the swallowing action; it will also bring chewed food to the taste buds of the tongue papillae to help a person in tasting the food. 33 Xerostomia alters the quantity and/or quality of saliva, which may cause difficulties in masticating, swallowing, speaking, and altered taste. 30 31 32 33 34 35 These conditions can affect OHRQoL negatively. This study showed that 70.6% of elders had low OHRQoL. The low OHRQoL of the majority of elders in this study was partly due to the low oral health awareness among Indonesians. Most Indonesians still believe that general health is more important than oral health.

A cross-sectional design was used in this study. Therefore, the limitation of this study lies in its failure to explain the causal relationship between xerostomia or NOP ≤ 5 and low OHRQoL. Another weakness of this research i.e. there is no assessment regarding the participants' cognitive, communication, and listening abilities beforehand. The inability of participants was only discovered during the study, thus causing the loss of participants. Further research must be performed with a sophisticated study design and sampling method to assure the representativeness of the sample of the population of elders living in Yogyakarta. Developing a simple OHRQoL assessment tool originating from Indonesia is necessary to facilitate its adjustment to the context of Indonesian people and its understanding by the elderly people at all levels of education, without neglecting the aspect of representativeness. According to Rai et al., to date there are no studies have been conducted to reveal oral conditions for low OHRQoL in Indonesia or worldwide. 46 This study will be a starting point and reference for further research so that we will get more complete data on oral conditions as risk factors for low OHRQoL. Those data are very important as a reference in doing better oral management for elderly patients so that they can improve their quality of life. These are the advantages and strengths of this study.

## Conclusions

Xerostomia and NOP ≤5 are the main risk factors that contribute to the occurrence of low OHRQoL in the elders living in Yogyakarta. Elders who suffer from xerostomia or NOP ≤5 have 2.5 times higher risks of experiencing low OHRQoL.
